# “Is the incidence of non-epithelial ovarian tumors on the rise? insights from a Tunisian tertiary center”

**DOI:** 10.3389/fonc.2025.1606243

**Published:** 2025-10-15

**Authors:** Ghada Abdelmoula, Mariem Garci, Nihed Abdessayed, Mehdi Makni, Amani Abdeljabbar, Wafa Babay, Nahla Ben Saada, Nabil Mathlouthi, Cyrine Belghith, Olfa Slimani

**Affiliations:** ^1^ Department A of Gynecology and Obstetrics, Charles Nicolle University Hospital of Tunis, Tunis, Tunisia; ^2^ Faculty of Medicine of Sousse, University of Sousse, Sousse, Tunisia; ^3^ Faculty of Medicine of Tunis, University of Tunis El Manar, Tunis, Tunisia; ^4^ Department of Anatomic Pathology, Farhat Hached University Hospital of Sousse, Sousse, Tunisia; ^5^ Laboratory of microorganisms and actives biomolecules, Faculty of sciences, University of Tunis El Manar, Tunis, Tunisia

**Keywords:** ovarian neoplasms, germ cell tumor, sex cord-stromal tumor, granulosa cell tumor, malignant < benign < pathology

## Abstract

**Introduction:**

Non-epithelial ovarian tumors (NEOTs), mainly germ cell and sex cord-stromal tumors, are rare entities that pose diagnostic and therapeutic challenges due to their heterogeneity and often nonspecific presentation. This study aimed to describe the epidemiological, clinical, pathological, and surgical characteristics of NEOTs managed at Charles Nicolle University Hospital, Tunis, over a five-year period.

**Materials and methods:**

We conducted a retrospective descriptive study including 48 patients operated for NEOTs between January 2020 and December 2024. Clinical, radiological, surgical, and pathological data were analyzed.

**Results:**

NEOTs represented 20.9% (48/229) of ovarian tumors. Median age at diagnosis was 35 years (IQR 28–51). Germ cell tumors accounted for 68.8% and sex cord-stromal tumors for 29.1%. Malignant tumors were rare (6.3%), all stage IA. Conservative surgery was performed in 56.2%, predominantly in germ cell tumors, while laparotomy was the main approach (87.5%). Compared with germ cell tumors, sex cord-stromal tumors occurred in older (median 51 vs. 30 years, p=0.003), more frequently postmenopausal patients (57.1% vs. 12.1%, p=0.003), and were more often >10 cm (61.5% vs. 25.8%, p=0.04). Postoperative complications occurred in 8.3%, and no recurrences were observed during follow-up.

**Conclusion:**

NEOTs, though rare, accounted for a relatively high proportion of ovarian tumors in our series. They were predominantly benign and diagnosed at an early stage, with favorable outcomes. Conservative surgery should be prioritized in young women to preserve fertility. This study represents the first Tunisian series addressing all histological subtypes of NEOTs and provides a reference for future multicenter research.

## Introduction

Non-epithelial ovarian tumors (NEOTs) are rare, with an incidence of fewer than 6 cases per 100,000 women per year (1). Their low prevalence and the scarcity of clinical and prognostic data pose major challenges for diagnosis and management (2). Unlike epithelial ovarian tumors, NEOTs arise from diverse precursor cells—germ cells, granulosa cells, thecal cells, and stromal fibroblasts—resulting in heterogeneous histological subtypes with distinct biological behaviors and therapeutic responses (1).

The deep anatomical location of the ovaries and the nonspecific nature of early symptoms often contribute to delayed diagnosis. Furthermore, the wide histological spectrum complicates both classification and therapeutic decision-making (3). In this context, accurate histopathological characterization and individualized treatment strategies are essential to optimize patient outcomes.

Despite advances in oncologic research, data on NEOTs remain scarce, particularly in North Africa. To our knowledge, no comprehensive national series has been published in Tunisia. This study therefore aims to describe the epidemiological, clinical, radiological, and pathological features of NEOTs managed in a tertiary center, to evaluate therapeutic approaches, and to compare our findings with the existing literature.

## Methods

This was a retrospective descriptive study conducted over a five-year period, from January 2020 to December 2024, at the Department of Obstetrics and Gynecology A of Charles Nicolle University Hospital in Tunis, Tunisia. During this period, 229 patients underwent surgery for an ovarian tumor. Of these, 48 consecutive cases were histologically confirmed as non-epithelial ovarian tumors (NEOTs) and were included in the analysis. Histological classification followed the 2020 World Health Organization (WHO) criteria for ovarian tumors.

**Figure 1:**
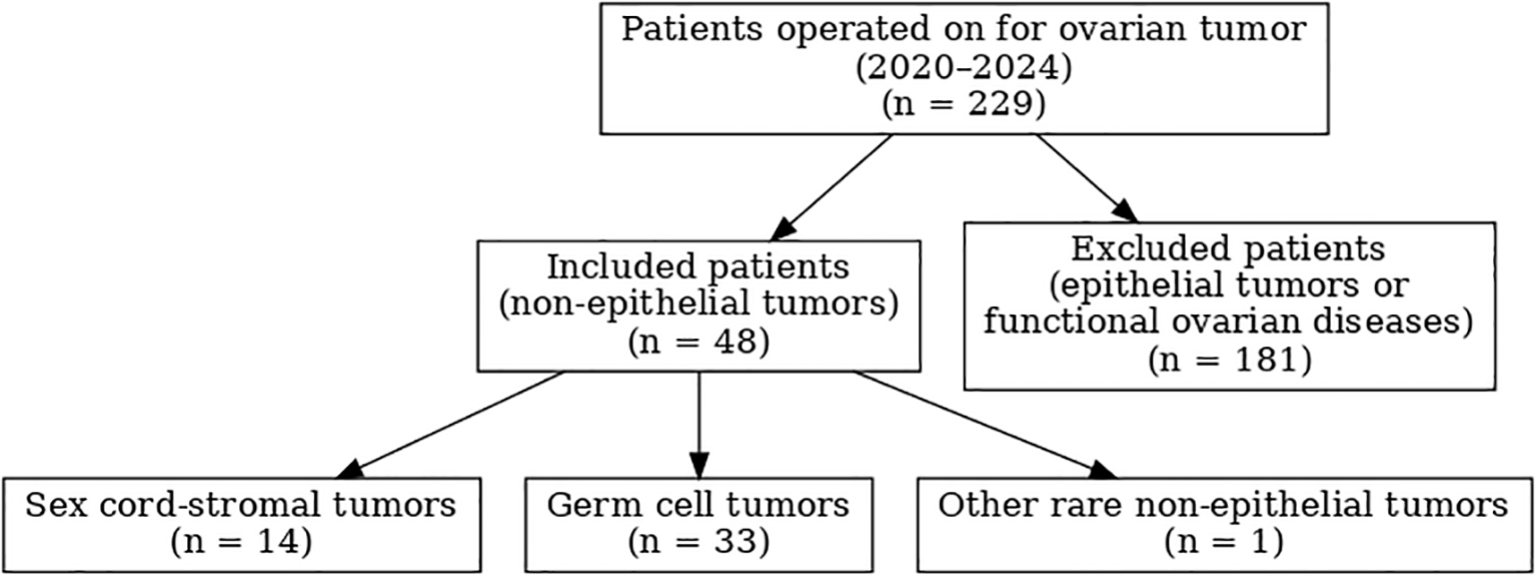
Flow chart of patient selection according to STROBE guidelines: 229 patients operated on for ovarian tumor between 2020 and 2024, including 48 with non-epithelial ovarian tumors.

Inclusion criteria comprised all patients operated on in our department during the study period with a histologically confirmed NEOT. Exclusion criteria were epithelial ovarian tumors, functional ovarian lesions such as functional cysts and endometriomas, absence of histological confirmation, and incomplete medical records. A patient selection flowchart, constructed in accordance with STROBE guidelines, is provided in **Figure 1**.

Data collection relied exclusively on medical records, including hospitalization files, operative notes, pathology reports, and imaging studies. No patient was re-contacted. Information was extracted using a standardized data collection form and encompassed epidemiological characteristics (age, parity, menopausal status, use of hormonal contraception, personal and family history of cancer), clinical presentation (symptoms at diagnosis, delay before consultation, physical examination findings), imaging findings (ultrasound, CT, MRI, with O-RADS classification when available), tumor markers (AFP, β-hCG, and LDH for germ cell tumors; inhibin B and AMH for granulosa cell tumors; CA-125 or HE4 when indicated), pathological features (macroscopic and microscopic findings, WHO 2020 classification, and FIGO staging for malignant cases), and therapeutic management (surgical approach, conservative vs. radical procedures, complementary staging interventions, adjuvant treatment). Postoperative outcomes were also recorded, including complications classified according to the Clavien–Dindo system whenever possible, length of hospital stay, duration of follow-up, and recurrence or survival status.

To ensure clarity and consistency, all variable definitions were standardized *a priori*. **Parity** was categorized as nulliparous (no prior delivery), pauciparous (1–2 deliveries), multiparous (3–4 deliveries), and grand multiparous (≥5 deliveries). **Tumor size** was classified as small (<5 cm), medium (5–10 cm), or large (>10 cm) based on the greatest diameter. **Tumor wall thickness** was defined macroscopically as thin (<3 mm) or thick (≥3 mm). These definitions were applied consistently throughout data collection and analysis.

Statistical analyses were performed using SPSS software, version 20 (IBM Corp., Armonk, NY, USA). Categorical variables were expressed as frequencies and percentages, and compared using the χ² test or Fisher’s exact test as appropriate. Continuous variables were tested for normality using the Shapiro–Wilk test. Normally distributed variables were expressed as mean ± standard deviation (SD) and compared with the Student t-test, whereas non-normally distributed variables were reported as median and interquartile range (IQR) and compared with the Mann–Whitney U test. For key estimates, 95% confidence intervals (CI) were calculated, and statistical significance was set at a p-value <0.05. Analyses were performed on available cases (complete case analysis), and missing data were explicitly reported.

This study was conducted in accordance with the Declaration of Helsinki. As it was based exclusively on anonymized retrospective data from medical records, formal ethical approval was not required under national regulations or institutional policy at Charles Nicolle University Hospital. Patient confidentiality was strictly maintained, and no identifying information was collected.

## Results

### Descriptive analysis

During the study period, 48 patients with non-epithelial ovarian tumors (NEOT) were included, representing 20.9% of all ovarian tumors operated on (n = 229). The median age at diagnosis was 35 years (IQR 28–51; range 17–71). The most affected age groups were 30–39 years (31.3%) and 20–29 years (22.9%). The majority of patients were married (70.8%), with a middle socio-economic level (60.4%).

A history of medical comorbidities was found in 22.9% of patients, mainly hypertension (16.7%) and diabetes (8.3%). Smoking was reported in 8.3%. Prior abdominal or pelvic surgery was noted in 18.8% of cases, most frequently cholecystectomy, hernia repair, and appendectomy. The mean age at menarche was 12.8 ± 1.4 years, with late menarche (>13 years) observed in 37.5%. Most women were premenopausal (75%), while 25% were postmenopausal, with a mean age at natural menopause of 51.2 ± 3.5 years. The median number of pregnancies was 2 (IQR 0–4), and 66.7% of patients had given birth. Infertility was reported in 4.2%. Contraceptive use was noted in 27.1%, predominantly intrauterine devices. No patient had received ovarian stimulation or hormone replacement therapy. A history of benign gynecological conditions was reported in 14.6%, and a family history of cancer in 8.3%.

The main demographic, reproductive, and clinical characteristics are summarized in **Table 1**.

**Table 1:** Baseline characteristics of patients with non-epithelial ovarian tumors (n=48).

Characteristic	n (%) or median (IQR)
Total patients	48 (100%)
Age, years	Median 35 (IQR 28–51); range 17–71
Marital status	Married 34 (70.8%); Single 12 (25.0%); Divorced 2 (4.2%)
Socio-economic level	Low 12 (25.0%); Middle 29 (60.4%); High 7 (14.6%)
Medical comorbidity (≥1)	11 (22.9%) — HTA 8 (16.7%); Diabetes 4 (8.3%)
Smoking	4 (8.3%)
Prior surgery	9 (18.8%) — cholecystectomy 3 (6.3%); hernia repair 2 (4.2%); appendectomy 2 (4.2%)
Menarche, years	Mean 12.8 ± 1.4 (range 10–17); late menarche (>13 yrs) 18 (37.5%)
Menopausal status	Premenopausal 36 (75.0%); Postmenopausal 12 (25.0%)
Gestity	Median 2 (IQR 0–4); pregnancies observed in 34 (70.9%) — paucigravida 19 (39.6%); multigravida 15 (31.3%)
Parity	Median 1 (IQR 0–5); gave birth 32 (66.7%) — pauciparous 26 (54.2%); multiparous 6 (12.5%); nulliparous 16 (33.3%)
Infertility	2 (4.2%)
Contraception	13 (27.1%) — IUD 9 (18.8%); oral contraception 2 (4.2%); tubal ligation 2 (4.2%)
Family history of cancer	4 (8.3%)
Presentation: symptomatic	42 (87.5%); asymptomatic 6 (12.5%)
Delay to consultation	Median 75 days (IQR 25–120; range 1 day–10 years)
Main circumstances of discovery	Abdomino-pelvic pain 19 (39.6%); Suspected torsion 10 (20.8%)
Clinical exam abnormal	21 (43.9%) — pelvic mass 9 (18.8%); abdominal tenderness 9 (18.8%); cul-de-sac finding 3 (6.3%)
Ultrasound detected tumor	45/48 (93.8%) — (N = 45 for subsequent US descriptors)
• Laterality (of 45)	Unilateral 42 (93.3%); Right 22 (48.9%); Left 20 (44.4%); Bilateral 3 (6.7%)
• Tumor size on US (of 45)	<5 cm 13 (28.9%); 5–10 cm 16 (35.6%); >10 cm 16 (35.6%)
• Wall	Thin 32/45 (71.1%); Thick 13/45 (28.9%)
• Cavity	Unilocular 29/45 (64.4%); Multilocular 16/45 (35.6%)
• Echostructure	Liquid 7/45 (15.6%); Solid 4/45 (8.9%); Mixed 34/45 (75.6%)
• Vegetations (of 45)	6 (13.3%)
CT performed	12/48 (25.0%)
MRI performed	21/48 (43.8%)
Tumor markers measured	23/48 (47.9%); elevated in 7/23 (30.4%)
Surgical approach	Laparotomy 42 (87.5%); Laparoscopy 6 (12.5%)
Initial surgical strategy	Radical 20 (41.7%); Conservative 27 (56.2%); Conservative then completion 1 (2.1%)
Frozen section performed	14 (29.2%)
FIGO staging performed	3 (6.3%) — all FIGO IA (these were the malignant cases)
Secondary (complementary) surgery	4 (8.3%) — unilateral adnexectomy 1; total hysterectomy + omentectomy 3
Macroscopic appearance	Cystic 21 (43.8%); Solid-cystic 18 (37.5%); Solid 9 (18.8%)
Histology	Benign 45 (93.8%); Malignant 3 (6.3%)
• Histological types	Germ cell tumors 33 (68.8%); Sex cord-stromal tumors 14 (29.2%); Other (mesothelial cyst) 1 (2.1%)
Perioperative complications	0 (0%)
Postoperative complications	4 (8.3%) — ischemic stroke 1; pelvic infection 1; sigmoid necrosis → colostomy 1; peritonitis 1
Recurrence during reported follow-up	0 (0%)

At presentation, 87.5% of patients were symptomatic. The median time to consultation was 75 days (IQR 25–120), and more than one-quarter consulted within the first month. Abdomino-pelvic pain was the most frequent symptom (39.6%), followed by acute presentations suggesting adnexal torsion (20.8%). Clinical examination was abnormal in 43.9% of cases, most commonly revealing an abdomino-pelvic mass or abdominal tenderness.

Ultrasound was contributive in 93.7% of patients. Tumors were unilateral in 93.3% of cases, equally distributed between right and left sides, and bilateral in 6.7%. The tumor size exceeded 10 cm in 35.6% of cases, with cystic, mixed, and solid echostructures observed in 15.6%, 75.6%, and 8.9%, respectively. Vegetations were detected in 13.3%. CT was performed in 25% of patients, mostly in emergency settings, and MRI in 43.8% for indeterminate or suspicious masses. Tumor markers were assessed in 47.9%, with elevated values (mainly CA-125) in 30.4%.

Surgery was the mainstay of treatment. Laparotomy was performed in 87.5% of patients and laparoscopy in 12.5%. Radical surgery was undertaken in 41.7%, conservative surgery in 56.2%, and conservative surgery followed by completion in 2.1%. Frozen section analysis was performed in 29.2%. FIGO staging procedures were carried out in three malignant cases, all stage IA. Secondary surgery was required in 8.3% of patients.

Macroscopically, tumors were cystic in 43.8%, mixed solid-cystic in 37.5%, and solid in 18.8%. Histopathological examination confirmed benign lesions in 93.7% and malignant tumors in 6.3%. Sex cord-stromal tumors accounted for 29.1%, germ cell tumors for 68.8%, and one case was diagnosed as a mesothelial cyst.

No perioperative complications were recorded. Postoperative morbidity occurred in four patients (8.3%), including ischemic stroke, pelvic infection, sigmoid necrosis requiring colostomy, and peritonitis. No recurrence was observed during the follow-up period.

### Comparative analysis

Comparative analysis was conducted between sex cord-stromal tumors (SCST, n = 14) and germ cell tumors (GCT, n = 33). Patients with SCST were significantly older than those with GCT, with a median age of 51 years versus 30 years (*p* = 0.003). Postmenopausal women were also more frequent in the SCST group (57.1% vs 12.1%, *p* = 0.003). A history of medical comorbidities was more frequent in SCST (42.9% vs 15.2%), although this difference did not reach statistical significance (*p* = 0.061).

All SCST patients were symptomatic, compared to 81.8% of those with GCT (p = 0.159). The median time to diagnosis was similar between the two groups. Abdomino-pelvic pain was the leading presenting complaint in both groups (50% in SCST vs 36.4% in GCT, *p* = 0.518). Physical examination findings were also comparable.

Regarding imaging, tumors larger than 10 cm were significantly more frequent in the SCST group compared with GCT (61.5% vs 25.8%, *p* = 0.04). No other significant differences were noted in ultrasound features such as wall thickness, multilocularity, echostructure, or presence of vegetations.

In terms of surgical management, laparotomy was the predominant approach in both groups. Bilateral adnexectomy was significantly more frequent in SCST compared to GCT (42.9% vs 6.1%, *p* = 0.005), while cystectomy was more often performed in GCT (66.7% vs 35.7%, *p* = 0.049). Secondary surgery was also more frequently required in SCST (28.6% vs 0%, *p* = 0.006). Other procedures, including unilateral adnexectomy, hysterectomy, or omentectomy, showed no significant differences between groups.

A detailed comparison between SCST and GCT is presented in **Table 2**.

**Table 2:** Comparison between germ cell tumors and sex cord–stromal tumors.

Variable	Total (N = 47)	TCS (n=14)	TG (n=33)	p-value
Age, years (median [IQR])	35 [28–51]	51 [38–57]	30 [27–40]	0.003
Socio-economic status				
Low	11 (23.4%)	5 (35.7%)	6 (18.2%)	0.263
Middle	29 (61.7%)	8 (57.1%)	21 (63.6%)	0.675
High	7 (14.9%)	1 (7.1%)	6 (18.2%)	0.657
Medical comorbidity	11 (23.4%)	6 (42.9%)	5 (15.2%)	0.061
Smoking	4 (8.5%)	0 (0%)	4 (12.1%)	0.302
Prior surgery	9 (19.1%)	5 (35.7%)	4 (12.1%)	0.102
Menarche, years (mean ± SD)	12.8 ± 1.4	13.4 ± 1.7	12.6 ± 1.2	0.071
Late menarche (>13 yrs)	18 (38.3%)	7 (50%)	11 (33.3%)	0.282
Menopausal status				
Premenopausal	35 (74.5%)	6 (42.9%)	29 (87.9%)	0.003
Postmenopausal	12 (25.5%)	8 (57.1%)	4 (12.1%)	
Gestity, median [IQR]	2 [0–4]	2 [1–5]	2 [0–4]	0.506
Parity, median [IQR]	1 [0–3]	2 [1–3]	1 [0–3]	0.303
Infertility history	2 (4.3%)	2 (14.3%)	0 (0%)	0.084
Contraception use	13 (27.7%)	3 (21.4%)	10 (30.3%)	0.726
Family history of cancer	4 (8.5%)	2 (14.3%)	2 (6.1%)	0.572
Symptomatic at presentation	41 (87.2%)	14 (100%)	27 (81.8%)	0.159
Delay to diagnosis, days (median [IQR])	75 [25–120]	60 [30–90]	90 [24–120]	0.389
Tumor size >10 cm (US)	16/44 (36.4%)	8/13 (61.5%)	8/31 (25.8%)	0.04
Unilocular cavity (US)	28/44 (63.6%)	11/13 (84.6%)	17/31 (54.8%)	0.089
Solid echostructure (US)	4/44 (9.1%)	3/13 (23.1%)	1/31 (3.2%)	0.071
Annexectomy bilateral	8 (17.0%)	6 (42.9%)	2 (6.1%)	0.005
Cystectomy	27 (57.4%)	5 (35.7%)	22 (66.7%)	0.049
Completion surgery	4 (8.5%)	4 (28.6%)	0 (0%)	0.006
Postoperative complications	4 (8.5%)	2 (14.3%)	2 (6.1%)	0.572

Postoperative outcomes were generally favorable. Nevertheless, four patients (8.3%) developed complications during the immediate postoperative period. One patient experienced an ischemic stroke on the second postoperative day, which required admission to the intensive care unit and was classified as a Clavien–Dindo grade IV event. Another patient developed a sigmoid necrosis complicated by peritonitis on postoperative day five; she underwent reoperation with colostomy, corresponding to a grade IIIb complication. A febrile genital infection occurred in one patient on postoperative day 24, successfully treated with intravenous antibiotics (grade II). Finally, a minor wound dehiscence was noted in one case and managed conservatively with local wound care (grade I). All patients recovered without long-term sequelae. The median hospital stay was longer in patients with complications compared to those with uneventful courses (12 days [IQR 10–16] versus 5 days [IQR 4–7]).

The median follow-up duration was 24 months (IQR 10–36; range 1–60 months). No tumor recurrence was observed during this period. However, given that several patients had only short follow-up due to inclusion until December 2024, these findings should be interpreted with caution, and longer surveillance is required to assess long-term outcomes.

## Discussion

Ovarian cancer remains a major global health concern, ranking as the eighth most common malignancy in women and accounting for 3.7% of new cases and 4.7% of cancer-related deaths in 2020 (4). While incidence rates have declined in Northern Europe and North America, they continue to rise in parts of Eastern Europe and Asia. In Tunisia, ovarian cancer was responsible for 192 deaths in 2020, corresponding to 0.31% of all deaths, with an age-adjusted mortality rate of 2.83 per 100,000 (5). Non-epithelial ovarian tumors are rare, representing approximately 10% of ovarian malignancies and with an estimated incidence of 0.25 per 100,000 (6–8). Their prevalence shows geographic variation, accounting for 5–6% of ovarian cancers in Europe, North America, and Oceania, but up to 9% in Asia and Central/South America (9). To date, no epidemiological data have been published in Tunisia. Interestingly, our study revealed a frequency of 20.96%, considerably higher than international reports, which may reflect genetic, geographic, or environmental factors specific to our population.

Age distribution strongly differentiated histological subtypes. The mean age for sex cord–stromal tumors in our cohort was 51 years, in line with Hamra et al. (49.8 years) (10) and Mamouni et al. (48 years) (11). Granulosa cell tumors followed the expected dichotomy, with younger onset for the juvenile type (32.5 years) and older for the adult type (49 years) (2, 12–14). Fibrotecomas occurred at a mean age of 53.4 years, comparable to prior series (2, 15). Two cases of sex cord tumors with annular tubules were diagnosed at a mean age of 40 years, consistent with Young et al. (16), and were not associated with Peutz–Jeghers syndrome. Germ cell tumors occurred at a mean age of 30 years, in agreement with earlier studies (2, 8, 11, 17, 18). Comparative analysis confirmed significantly younger ages for germ cell tumors compared with sex cord–stromal tumors (p = 0.003).

Genetic predispositions are rare but clinically relevant. While up to 23% of adnexal malignancies are linked to hereditary syndromes, particularly BRCA mutations and Lynch syndrome, these predominantly concern epithelial tumors (19, 20). Non-epithelial tumors may occur in specific hereditary contexts such as DICER1 mutations (21), Peutz–Jeghers syndrome (22), or rhabdoid tumor predisposition (23). None of our patients reported such associations.

Reproductive factors also showed distinct profiles. Menarche occurred at a mean age of 12.8 years, consistent with regional cohorts (8, 17, 24). One quarter of patients were postmenopausal, with higher rates among sex cord–stromal tumors than germ cell tumors (57.1% vs. 12.1%; p = 0.003), in line with published data (14, 25, 26). Contraception was used by 27.1%, most often intrauterine devices, while oral contraceptives were uncommon. Although oral contraception reduces the risk of epithelial ovarian cancer (27), its impact on non-epithelial tumors appears negligible (28). Parity distribution differed from Moroccan series (8, 13, 25), with the majority of our patients being pauciparous (54.2%), though parity showed no protective effect, consistent with large epidemiological studies (29, 30). Infertility was reported in 4.2% of cases, similar to Indian series (31), and may be explained by inhibin-mediated ovulatory dysfunction (32).

The mean delay to diagnosis was 2.5 months, shorter than in Moroccan series (8, 13) but longer than in Norris et al. (33). This likely reflects better access to imaging and specialist consultations in recent years, though nonspecific symptoms still contribute to delays. Abdominopelvic pain was the most common symptom (39.6%), albeit lower than reported elsewhere (8, 10, 11, 33, 34). Torsion was suspected in 20.8% of our cases, higher than prior reports (33), possibly due to improved imaging. Incidental discovery accounted for 12.5%, reflecting the growing role of routine imaging. Other symptoms, including hypogastric heaviness, compressive signs, and metrorrhagia, were infrequent. Notably, more than half of patients (56.3%) had normal clinical examinations, underscoring the limitations of physical examination alone and the critical role of imaging (35).

Ultrasound was performed in nearly all patients (95%) and revealed predominantly mixed solid–cystic morphology (75.6%). Sex cord–stromal tumors were more often larger than 10 cm compared with germ cell tumors (61.5% vs. 25.8%, p = 0.04), in line with literature (8, 11, 35). MRI was increasingly used (43.8%), surpassing CT (25%), highlighting its role in preoperative characterization (36). Biomarkers contributed selectively: inhibin B proved valuable in follow-up of granulosa tumors (37), while CA-125 showed poor sensitivity (38). AFP and β-hCG remain the most relevant markers in germ cell tumors (39), whereas LDH was underutilized in our cohort despite its diagnostic utility (38).

Surgical management reflected histological subtype and reproductive considerations. Overall, 93.3% of tumors were unilateral, consistent with prior series (8, 40). Conservative surgery was performed in 56.2% of cases, more frequently in germ cell tumors (66.7%) than in sex cord–stromal tumors (35.7%; p = 0.049), in line with international data (10). Guidelines recommend fertility-sparing surgery for localized disease in young patients, with radical surgery reserved for postmenopausal women or advanced stages (41). In our series, all malignant cases were diagnosed at FIGO stage I, consistent with previous reports emphasizing early-stage presentation (8, 42).

Our study has several strengths. It represents the first Tunisian report on the overall frequency and clinicopathological features of non-epithelial ovarian tumors, providing novel national data. The five-year observation period and the inclusion of all consecutive cases enhance reliability. Detailed clinicopathological characterization and the emphasis on fertility-sparing surgery in young patients are notable contributions. However, limitations include the retrospective design, modest sample size, incomplete biomarker testing, and single-center recruitment, which restrict generalizability. Despite these constraints, our findings provide valuable insight into the epidemiology and management of non-epithelial ovarian tumors in Tunisia and establish a foundation for future multicenter and prospective studies.

## Conclusion

Non-epithelial ovarian tumors constitute a heterogeneous and rare group of neoplasms, whose clinical and epidemiological patterns differ markedly from epithelial ovarian cancers. Our study, the first of its kind in Tunisia, provides original insights by establishing their frequency and describing their main epidemiological, clinical, radiological, surgical, and pathological characteristics within a well-defined population. The high proportion of non-epithelial tumors observed in our cohort, compared with international series, may reflect specific local or genetic factors and underscores the importance of regional data.

The predominance of early-stage diagnoses and the feasibility of fertility-sparing approaches in young women highlight the potential for favorable outcomes when management is timely and adapted to histological subtype. However, the retrospective nature of the study, the limited sample size, and the single-center design restrict the generalizability of our results.

Despite these limitations, this work represents a novel contribution to the national literature and provides a foundation for multicenter and prospective studies aimed at improving diagnostic pathways, refining surgical strategies, and ultimately optimizing patient outcomes in the context of rare ovarian tumors.

### What is already know on this topic

1. Histological Diversity and Diagnosis: Non-epithelial ovarian tumors exhibit a wide range of histological types, including germ cell tumors, sex cord-stromal tumors, and other rare subtypes. Due to this diversity, accurate diagnosis often requires a combination of imaging techniques, tumor markers, and histopathological examination.
2. Surgical Management and Adjuvant Therapy: Surgical resection is the primary treatment for non-epithelial ovarian tumors, aiming for complete tumor removal. Depending on the tumor type and stage, patients may receive adjuvant chemotherapy, commonly using platinum-based regimens. The role of chemotherapy and other adjuvant therapies varies according to the specific histological subtype and clinical stage of the disease.

### What this study adds

- This is the first study from Tunisia to comprehensively assess the frequency and clinicopathological features of non-epithelial ovarian tumors across all histological subtypes.
- It demonstrates a higher frequency of these tumors compared to international data, highlighting possible geographic or population-specific factors.
- The study confirms that most cases are diagnosed at an early stage and that fertility-sparing surgery is feasible and effective in young women.
- It provides a national reference for future multicenter or prospective investigations on this rare group of ovarian tumors.

## Data Availability

The original contributions presented in the study are included in the article/supplementary material. Further inquiries can be directed to the corresponding author.
